# Description of a new species of the genus *Monelata* Förster, 1856 from China (Hymenoptera, Diapriidae)

**DOI:** 10.3897/zookeys.574.7628

**Published:** 2016-03-28

**Authors:** Zi Hou, Zai-fu Xu

**Affiliations:** 1 Department of Entomology, South China Agricultural University, Guangzhou 510640, China

**Keywords:** Hymenoptera, Diapriinae, *Monelata*, new species, Oriental Region, China

## Abstract

A new species of the genus *Monelata* Förster, 1856, *Monelata truncata* **sp. n.**, is described and illustrated from Yunnan Province, China. This is the third Oriental species assigned to this genus. A key to Oriental species of the genus is provided.

## Introduction

*Monelata* Förster, 1856 belongs to the tribe Diapriini of the subfamily Diapriinae (Hymenoptera, Diapriidae). Currently the genus includes sixteen species, of which nine are found in the Palearctic Region, one in the Palearctic and Nearctic Regions, two each in the Nearctic, Afrotropical and Oriental Regions (Johnson 1992; Masner and Garcia 2002; Rajmohana 2006). Little is known of their biology, but it is thought that they are probably parasitizing some Diptera (Masner and Garcia 2002).

In the Oriental Region, Huggert (1982) found one new female species in India, *Monelata incisipennis* Huggert, 1982. Rajmohana and Narendran (2000) reported the second new species, also from India, *Monelata completa* Rajmohana & Narendran, 2000. Liu and Xu (2012) founded the males of *Monelata incisipennis* Huggert, 1982 from China. In this paper, one new species from Yunnan Province, China is described, *Monelata truncata* sp. n.. A key to the Oriental species of this genus is provided.

## Materials and methods

Specimens were examined under a Leica MZ12.5 stereomicroscope. All photos were taken with a digital camera (Cool SNAP) attached to the Zeiss Stemi 2000-CS stereomicroscope and processed by using Image-Pro Plus software. Some holotypes of *Monelata* deposited in the Natural History Museum, London, UK (BMNH) were checked for this study.

Morphological terminology follows Masner and Garcia (2002). In the descriptions, abbreviations are as follows:

**A1**, **A2**, … = the first, second, .... antennal segments, respectively; OL = the distance between inner edge of lateral ocellus and median ocellus; OOL = the shortest distance between lateral ocellus and compound eye; POL = the shortest distance between inner margins of two posterior ocelli; S2 = the second metasomal sterite. T2 = the second metasomal tergite. Measurements reported are relative, except for body length (head to abdominal tip, excluded the antennae and ovipositor) and fore wing length.

## Taxonomy

### Genus Monelata

Taxon classificationAnimaliaHymenopteraDiapriidae

Förster, 1856

Monelata Förster, 1856: 123. Type species: Diapria parvula Nees von Esenbeck, designated by Ashmead (1893).
Monelata Förster: Ashmead 1893: 407; Dalla Torre 1898: 432; Kieffer 1910: 698; 1912: 5; 1916: 8; Prisner 1953: 452; Muesebeck and Walkley 1951: 676; 1956: 371; Pschorn-Walcher 1956: 58; Masner and Sundholm 1959: 165; Kozlov 1978: 594; Muesebeck 1979: 1143; Szabó 1979: 273; Masner and Garcia 2002: 93; Rajmohana 2006: 57.
Monelata For detailed generic synonymy see Johnson (1992).

#### Diagnosis.

Body length 0.9–1.5 mm, smooth and shining. Female antenna 13-segmented, with A13 remarkably clavate. Male antenna 14-segmented, with A4 not sexually modified. Cervix of pronotum densely hairy, dense pronotal and propleural cushions confluent into complete hairy collar ventrally and dorsally. Notauli absent. Anterior scutellar pit absent. Metapleuron densely covered with appressed pilosity, pilosity continuing on metasternum, completely surrounding hind coxae. Propodeum and petiole entirely carpeted with dense semi-hyaline pilosity. T2 with pilosity at base. S2 with large and dense anterior cushion.

#### Distribution.

Afrotropical, Nearctic, Palearctic and Oriental Regions (Rajmohana 2006; Johnson et al. 2015).

#### Key to Oriental species of *Monelata*

**Table:** 

1	Fore wing with apical margin deeply incised	***Monelata incisipennis* Huggert**
–	Fore wing with apical margin round or truncate	**2**
2	Forewing with apical margin round; head in dorsal view nearly as long as wide; mesosoma brown to reddish-brown	***Monelata completa* Rajmohana & Narendran**
–	Forewing with apical margin truncate (Fig. 3); head in dorsal view distinctly transverse; mesosoma black	***Monelata truncata* sp. n.**

### Monelata incisipennis

Taxon classificationAnimaliaHymenopteraDiapriidae

Huggert, 1982

Monelata incisipennis Huggert, 1982: 183.
Monelata incisipennis Huggert: Rajmohana 2006: 57; Liu and Xu 2012: 460.

#### Material examined.

1 ♀, CHINA: Guangdong, Nankunshan, 2011.IX.27–29, Zai-fu Xu (SCAU); 1 ♂, Hainan, Yinggeling, 2008.XI.16-20, Ya-li Tang (SCAU); 1 ♂, Guangxi, Maoershan, 2005.VIII.2-10, Bin Xiao (SCAU); 92 ♀♀ 48 ♂♂, Yunnan, Zhaotong, Yongshan, Huanghua, 2012.VIII–X, Shi-wen Yang (SCAU).

#### Biology.

Unknown.

#### Distribution.

China (Guangdong, Hainan, Guangxi, Yunnan); India (Rajmohana 2006).

### Monelata truncata

Taxon classificationAnimaliaHymenopteraDiapriidae

Hou & Xu sp. n.

http://zoobank.org/2B503D40-A92F-4031-9730-8344634E40E4

Figs 1–3 , 4–5

#### Material examined.

Holotype: ♀, CHINA: Yunnan, Zhaotong, Yongshan, Huanghua, 2012.VIII–X, Shi-wen Yang (SCAU). Paratypes: 22 ♀♀ 35 ♂♂, Yunnan, Zhaotong, Yongshan, Huanghua, 2012.VIII–X, Shi-wen Yang (SCAU).

#### Description.

**Figures 1–3.**
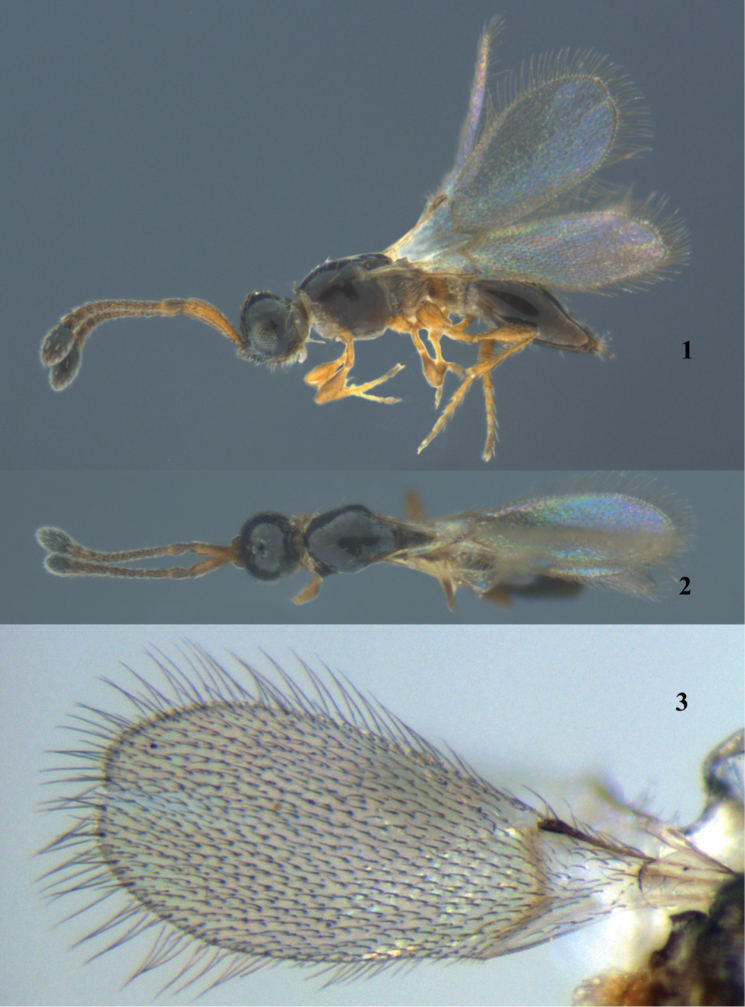
*Monelata truncata* sp. n., ♀, holotype, habitus. **1** Lateral view **2** dorsal view **3** fore wing.

Holotype. *Female* (Figs 1–3). Body length 0.9 mm. Fore wing length 0.8 mm.

*Color*. Head black. Antenna dark brown, with A13 black. Mesosoma and metasoma black. Petiole and legs brown. Fore and hind wings hyaline, with veins brown.

*Head*. Head in dorsal view transverse, 0.75 times as long as wide. Relative proportion of length to width of antennal segment as follows: A1 (10.5 : 2.2); A2 (3.5 : 1.8); A3 (1.6 : 1.3); A4 (1.2 : 1.3); A5 (1.2 : 1.3); A6 (1.2 : 1.3); A7 (1.2 : 1.3); A8 (1.3 : 1.4); A9 (1.4 : 1.5); A10 (1.6 : 1.6); A11 (1.8 : 2.0); A12 (2.2 : 2.3); A13 (7.5 : 4.5). A1 slender, cylindrical, unarmed apically. A10 to A13 gradually enlarged. A13 ovoid, remarkably clavate. Eye oval, 1.5 times as long as wide, 1.5 times as long as malar space. Posterior orbit of eye not sinuate. POL : OOL : OL = 1.5 : 4.0 : 1.5.

*Mesosoma*. Mesosoma as wide as head. Cervix densely hairy, dense pronotal and propleural cushions confluent into complete hairy collar ventrally and dorsally. Mesoscutum smooth, with three pairs of setae. Scutellar disc slightly converging posteriorly. Mesopleuron smooth. Metanotum with a median keel. Metapleuron covered with long hairs. Propodeum elongate, with a distinct median keel, pointed anteriorly. Posterior margin of propodeum excavate. Fore wing elongate, distinctly longer than mesosoma and metasoma; apical margin truncate (Fig. 3); apical margin with long fringes, 1/3 of wing width. Hind wing narrow, with fringes distinctly longer than wing width. Legs long and slender.

*Metasoma*. Petiole cylindrical, 1.5 times as long as wide. Petiole covered by dense, translucent, elongate setae. T2 enlarged, cover 0.8 length of gaster, 1.7 times as long as wide.

**Figures 4–5.**
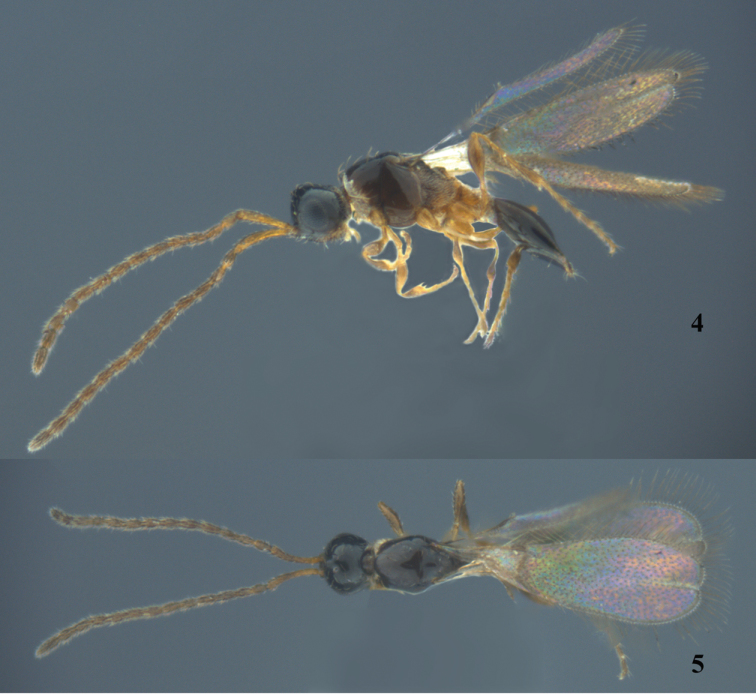
*Monelata truncata* sp. n., ♂, paratype, habitus. **4** Lateral view **5** dorsal view.

*Male* (Figs 4, 5). Body length 0.8 mm. Fore wing length 0.7 mm. Antenna fully brown. Relative proportion of length to width of antennal segment as follows: A1 (11.0 : 2.0); A2 (3.2 : 1.8); A3 (3.8 : 1.9); A4 (3.8 : 1.9); A5 (3.8 : 2.0); A6 (3.8 : 2.0); A7 (3.8 : 2.0); A8 (3.8 : 2.0); A9 (3.8 : 2.0); A10 (3.8 : 2.0); A11 (3.8 : 2.0); A12 (4.0 : 2.1); A13 (4.0 : 2.1); A14 (5.0 : 2.1). Eye 1.4 times as long as wide, 2.3 times as long as malar space. POL : OOL : OL = 1.5 : 4.0 : 1.3. A4 not sexually modified. Other characteristics are similar to females.

#### Variation.

*Females*. Body length 0.8–1.0 mm. Fore wing length 0.8–0.9 mm. *Males*. Body length 0.7–0.8 mm. Fore wing length 0.6–0.7 mm.

#### Distribution.

China (Yunnan).

#### Etymology.

The species name “*truncata*” is based on the truncate apical margin of fore wing.

#### Remarks.

This is the third species of the genus *Monelata* in the Oriental Region, but it can be separated from the other two Oriental species, *Monelata incisipennis* Huggert and *Monelata completa* Rajmohana & Narendran by the following characteristics: fore wing with apical margin truncate (deeply incised in *Monelata incisipennis*, and round in *Monelata completa*); head in dorsal view distinctly transverse (nearly as long as wide in the latter two); and mesosoma black (reddish-brown to brown in the latter two).

According to the key and figure of Nixon (1980), *Monelata solida* (Thomson, 1858) in the Palearctic Region is “forewing faintly to hardly emarginate at apex”, the new species is similar to *Monelata solida*. But after we checked the holotype of *Monelata solida* (Holotype ♀, BMNH No. 9.688) in London, the apical margin of fore wing of *Monelata solida* is round as *Monelata completa*. The new species also can be separated from *Monelata solida* by head in dorsal view distinctly transverse (slightly wider than long in *Monelata solida*); female A7–A12 slightly wider than long, or as wide as long (distinctly wider than long in *Monelata solida*); A12 1.67 times as long as wide (2.27 times in *Monelata solida*).

## Supplementary Material

XML Treatment for Monelata

XML Treatment for Monelata incisipennis

XML Treatment for Monelata truncata
